# The Book World of Medicine and Science

**Published:** 1907-06-01

**Authors:** 


### The Book World of Medicine and Science.

Metabolism and Practical Medicine. By many authors; edited by Carl von Noorden, Professor of the First University Medical Clinic, Vienna; English issue under the editorship of I. Walker Hall, Professor of Pathology, University College, Bristol; Pathologist to the Bristol Infirmary. (London: William Heinemann. Small 4to. 3 volumes, together price £2 12s. 6d. net. Vol. I., pp. 452. Vol. II., pp. 525. Vol. III., to be issued shortly.)

We congratulate Professor I. Walker Hall upon his English edition of Professor Carl von Noorden's well-known text-book. He has obtained the services of excellent translators, and although so monumental a work upon so complex a subject can scarcely be described as easy reading, the subjects dealt with have almost without exception been made as clear as possible by expressing the sense rather than the wording of the German. This is always a most important matter in making an English edition of a German work, and we think that Professor Walker Hall and his collaborators have succeeded excellently. The long German sentences have been broken up, and the compound adjectives have been divided and re-arranged in a way that has required much skill. Difficult though the subject matter is to those not already familiar with it, we are sure that this is not the fault of the English in the edition which is before us.

We are rather sorry that Professor von Noorden tends to be polemic in the chapters he himself has written; but upon the whole the writing is level-headed. If there is one fault in the German edition it is that Continental papers are fully given, whilst those of English and American authors are largely overlooked; this fault has been greatly improved by Professor Walker Hall in his English edition. The book should be in every medical reference library and in every chemico-pathological library; it is indispensable to those engaged in research. We think that every consulting physician should have a copy, to which he will be constantly referring; but those who have been in practice for some time will probably feel that it is too great a labour to try and become familiar with all this recent work. It would be very difficult to boil down the contents of these three volumes into one small handbook, and meanwhile the books before us are quite the best for those who wish to gain an insight into metabolism in relation to practical medicine.

Volume I. deals with the physiology of metabolism; that is to say, with our knowledge of the metabolic processes of healthy persons. It is translated from the German of Adolf Magnus-Levy, of Berlin. The chapters upon the foodstuffs, digestion and absorption, the fate of the foodstuffs in the tissues, the total energy exchange, the respiratory quotient, nitrogenous metabolism in general, and so on, are perhaps chiefly of interest to the physiologist, from whom the physician is content to learn the kernel of each question discussed; there are, however, other chapters which are of essential interest to the physician, to the gynæcologist, and possibly to the surgeon—namely, those upon the influence of climatic and other conditions upon the minimal metabolism, and of menstruation, pregnancy, and castration; the *rôle* of water in metabolism; the metabolism of mineral substances, sodium chloride, phosphorus, calcium, magnesium, the halogens, the alkalis; and metabolism in old age.

Volume II., written by von Noorden, Kraus, Schmidt, Weintraud, Mathers, and Strauss, deals with the pathology of metabolism in hunger and chronic starvation; in over-feeding; in fever and infection; in stomach diseases; in intestinal diseases; in diseases of the liver, respiration, circulation, blood, and kidneys.

Volume III. is not yet before us, but is to be a continuation of the pathology of metabolism, including diabetes mellitus, and general pathological conditions. We hope that, in addition to the general index of subjects, there will be an alphabetical index of all the authors referred to; there is none such in the first two volumes, and we miss it very much.

Tics and Their Treatment. By Henry Meige and E. Feindel, with a preface by Professor Brissand. Translated and edited by S. A. K. Wilson, M.A., M.B., B.Sc. (Published by Mr. Sidney Appleton, London. 8vo., pp. 386. No illustrations. Price 9s. net.)

This book is a masterpiece of its kind, dealing with tics of all sorts from every point of view. It is, however, a volume which we think will find its place in the libraries of hospitals, nerve specialists, and alienists, rather than in those of general practitioners. It is clearly written, full of details, well indexed and printed, and has a compilation of the literature upon the subject occupying 29 pages. It begins with a lucid account of a patient who not only suffered from a multitude of different tics, but had also the power of giving a graphic description of the origin, cause, and effects of each of them. It then proceeds to discuss at great length the definition of tics, distinguishing them clearly from spasms, tremors, simple habits good or bad, stereotyped acts, and so forth. The authors deprecate the term tic douloureaux, which has led to much confusion, seeing that tics are all motor phenomena, and that tic douloureux is not a tic at all. They lay stress upon the facts that only predisposed persons suffer from tics; that tics are essentially cortical in origin; that they are multifarious in kind; that they must have no reflex origin, or else they are spasms and not tics; that they are related to mental derangements. The authors' arguments are all clearly expressed, and we agree with them in the main; they give many illustrative cases which add much interest. The description of the different varieties of tic—facial, auditory, nictitating, sniffing, sucking, licking, and biting tics, tics of the neck, trunk, arm, shoulder, legs, tics of writing, spitting, swallowing, vomiting, wind-sucking, snoring, sniffing, blowing, whistling, coughing, sobbing, and hiccoughing, tics of speech—is excellent. Their differential diagnosis is discussed, and finally there are two chapters upon treatment; these are only 47 pages of the whole, but they are the chapters which will appeal most to the practitioner. The authors discuss every form of treatment that has been tried; they deprecate operative measures of all sorts, whether for spasmodic torticollis, or for any other tic, and have themselves observed the very greatest benefit from careful treatment by exercises, mirror drill, and re-education of the motor-centres, all of which are described in detail.

